# Evidence for direct use of terminal organ for spermatophore transfer in giant squid, *Architeuthis dux*

**DOI:** 10.17912/micropub.biology.001476

**Published:** 2025-04-04

**Authors:** Seiji Sasai, Yoshiaki Tanaka, Noritaka Hirohashi

**Affiliations:** 1 Echizen-Matsushima Aquarium, Sakai, Fukui Japan; 2 Shimane Aquarium, Hamada, Shimane Japan; 3 Shimane University, Matsue, Shimane, Japan

## Abstract

During mating, males of most cephalopods use a modified arm, known as a hectocotylus, to transfer spermatophores into the female. However, a long-standing enigma has been whether some deep-sea squids use a terminal organ (TO), similar to a penis, for direct spermatophore transfer, as suggested by anatomical observations. Here, we present evidence supporting this hypothesis in the giant squid, *Architeuthis dux* . Two male squids in the moribund condition were discovered in shallow water, with their TOs passing through their own funnels and being able of active movement, a behavior previously observed in *Pholidoteuthis adami* in deep water.

**Figure 1. The male giant squids stranded on the coasts of the Sea of Japan:**
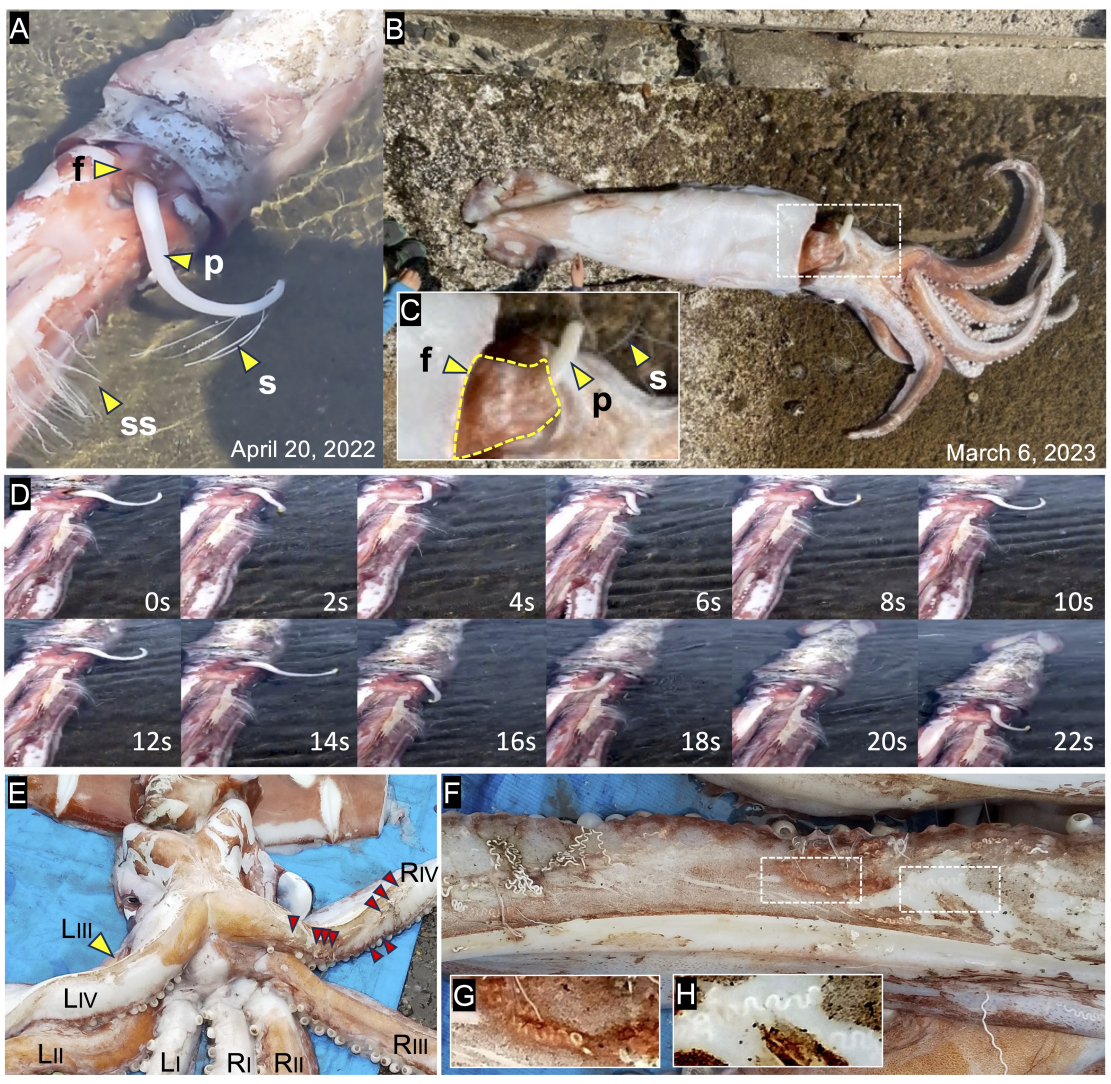
**A-C,** Males were found with their ventral side up and their terminal organs (p) being passed through the funnels (f). They were either still barely alive (A) or already dead (B) when observed. S, spermatophore; SS, spermatangium. **D,** Time-lapse images represent the movements of the terminal organ. **E-H,** A view of the arm crown from ventral side of the Fukui specimen (A) with spermatangia embedded under the skin (G) or intramuscularly (H) on the right fourth (RIV) arm. Red arrows point embedded spermatangia.

## Description

The morphology of male genitalia has diverged rapidly and intensively across animal kingdom (Eberhard, 1985; Arnqvist, 1998; Eberhard, 2009). This divergence may have occurred as a result of powerful evolutionary forces, known as sexually antagonistic coevolution and cryptic female choice, both of which are central to sexual selection (Hosken & Stockley, 2004; Eberhard, 2010). These hypotheses are feasible in promiscuous species because the primary role of male genitalia is to deliver sperm close to female gametes or the sites of sperm reservoir within the female, providing greater opportunities for sexual conflict to occur. However, deep-sea squids generally suffer from partner scarcely due to low population density. Therefore, both intrasexual and intersexual competition for mating are unlikely to occur. Curiously, in some deep-sea squids, there is ample anatomical evidence for the use of male genitalia, with direct transport of sperm capsules (spermatophores) into the female instead of passing through a hectocotylus (Nesis, 1995; Jackson & Jackson, 2004; Hoving & Laptikhovsky, 2007; Hoving et al., 2010a, 2010b). Because these changes are only reported in deep-sea squids among cephalopods, and they are not necessarily phylogenetically related, shared extreme environments may confer convergent functional and behavioral alternations in copulation (Arkhipkin & Laptikhovsky 2010; Hoving et al., 2014; Marian, 2014). Therefore, it is possible that the morphology and function of male squid genitalia have evolved due to selective forces for environmental adaptations rather than sexual conflict. However, there is currently no direct evidence to support this imaginal mating behavior, primary due to huge challenges in underwater observations and validation through capture. Nevertheless, the most compelling evidence obtained so far was the ROV recording, by which a pair of *Pholidoteuthis adami* , in an anti-parallel position, possibly mating by using the TO to penetrate through the funnel (Hoving & Vecchione, 2012). The giant squid, *A. dux* , may have a mating strategy similar to that of P *. adami* based on several morphological characteristics, such as an externally stretchable TO and implanted spermatangia under the male's skin within the reach of a TO (increasing the likelihood of self-implantation). We investigated two dying males that were found in shallow waters near the coasts of the Sea of Japan, which are known as hot spots for giant squid stranding (Kubodera et al., 2018); one in Obama, Fukui on April 20, 2022, 132 cm in mantle length, ca. 80 kg in body weight and the other in Chibu-Mura, Shimane on March 6, 2023, the mantle length unknown. In both cases, the specimens were filmed first by citizens and then by the television crews. The videos captured males passing their TOs through their own funnels ( Fig. 1A- C). Notably, in the Fukui specimen, the male wiggled its TO, while the other body parts remained motionless ( Fig. 1D, Supplemental movies). Along with these movements, spermatophores were expelled ( Fig. 1A, s), and there was a large number of shed spermatophores (spermatangia) adhered to the arms ( Fig. 1A, ss). The next day, we were able to access the dead specimen in Fukui and found that many spermatangia were deeply embedded under the skin of the right IV-arm ( Fig. 1E- 1H), which coincided with the sites where spermatangia were found adhered the day before ( Fig. 1A, ss). All of these observations were consistent with previous reports on the speculative views of *A. dux* reproduction (Norman & Lu, 1997; Hoving et al., 2004; Roper & Shea, 2013; Murai et al., 2021). As suggested in a previous report (Marian, 2014), we assume that the spermatophores likely have an intrinsic mechanism that allows them to penetrate the skin or even muscle. What advantage, if any, is conferred by the TO going through the funnel? One possible reason for this behavior would be that the movements of the funnel involved in the position of the TO. The TO in *P. adami* also went through the funnel, and the copulation took longer than expected (Hoving & Vecchione, 2012). Maybe the functional morphology of "TO through funnel" is about holding steady for a long copulation, and "TO outside the funnel" is for a more rapid response. Additionally, the funnel, with its fast-acting contractile muscles, may play a role in expelling or jetting the spermatophores into the female skin. A drastic change in mating patterns among deep-water squids may have significant causes and consequences. Lastly, we know so little about deep sea reproduction in cephalopods that anything is possible, awaiting further exploration in uncharted areas of research.

## Methods

With the power of social networking services, we collected information about stranding of unidentified squids, particularly giant-sized squids, along the coasts of the Sea of Japan. We have also distributed a "wanted flyer" similar to the one issued by Frederick Aldrich in 1988 (https://en.wikipedia.org/wiki/List_of_giant_squid_specimens_and_sightings) to aquariums, museums, and fisheries cooperatives located on the Sea of Japan side. Because the giant squid is one of the most important and popular exhibits in aquariums, and citizens are often the first to discover it, we also took part in the outreach activities for the aquarium exhibition. In line with this, we provided information and comments on the frequent strandings of deep-sea creatures to media sources. As a result of these mutually cooperative relationships, precious specimens have become available on the top of laboratory bench.

Data Availability

Videos are available on figshare at https://figshare.com/articles/media/A_giant_squid/27852069 
